# Time Trends in Prevalence of Chronic Diseases and Multimorbidity Not Only due to Aging: Data from General Practices and Health Surveys

**DOI:** 10.1371/journal.pone.0160264

**Published:** 2016-08-02

**Authors:** Sandra H van Oostrom, Ronald Gijsen, Irina Stirbu, Joke C Korevaar, Francois G Schellevis, H. Susan J Picavet, Nancy Hoeymans

**Affiliations:** 1 Centre for Nutrition, Prevention and Health Services, National Institute for Public Health and the Environment, Bilthoven, the Netherlands; 2 Centre for Health and Society, National Institute for Public Health and the Environment, Bilthoven, the Netherlands; 3 Netherlands Institute for Health Services Research (NIVEL), Utrecht, the Netherlands; 4 Department of General Practice and Elderly Care Medicine /EMGO Institute for health and care research, VU University Medical Centre, Amsterdam, the Netherlands; University of Brescia, ITALY

## Abstract

**Introduction:**

Chronic diseases and multimorbidity are common and expected to rise over the coming years. The objective of this study is to examine the time trend in the prevalence of chronic diseases and multimorbidity over the period 2001 till 2011 in the Netherlands, and the extent to which this can be ascribed to the aging of the population.

**Methods:**

Monitoring study, using two data sources: 1) medical records of patients listed in a nationally representative network of general practices over the period 2002–2011, and 2) national health interview surveys over the period 2001–2011. Regression models were used to study trends in the prevalence-rates over time, with and without standardization for age.

**Results:**

An increase from 34.9% to 41.8% (p<0.01) in the prevalence of chronic diseases was observed in the general practice registration over the period 2004–2011 and from 41.0% to 46.6% (p<0.01) based on self-reported diseases over the period 2001–2011. Multimorbidity increased from 12.7% to 16.2% (p<0.01) and from 14.3% to 17.5% (p<0.01), respectively. Aging of the population explained part of these trends: about one-fifth based on general practice data, and one-third for chronic diseases and half of the trend for multimorbidity based on health surveys.

**Conclusions:**

The prevalence of chronic diseases and multimorbidity increased over the period 2001–2011. Aging of the population only explained part of the increase, implying that other factors such as health care and society-related developments are responsible for a substantial part of this rise.

## Introduction

In western societies the prevalence of chronic diseases is increasing due to the rapid aging of the population and the greater longevity of people with chronic conditions [1]. Along with an increase in the number of those with specific diseases, the prevalence of multimorbidity, i.e. the presence of multiple diseases in the same individual, is rising [2]. For the US, a rise in the number of those with two or more chronic diseases over the period 2001–2010 was shown based on data from health surveys and data from health care insurance organisations [3–5]. This rise and the expectation of a further increase in the prevalence of chronic diseases [6] is a challenge for policymakers, since it has important implications for future health care policy and resource allocation [7]. Several countries are planning to reform their health care systems in order to be better equipped to manage the growing proportion of older people with chronic conditions [8].

In addition to the impact of the aging of the population, several other factors may have had an effect on the rise of the estimated prevalences of chronic diseases. These are, in particular, earlier and improved detection of diseases, advances in medical treatments, and changing lifestyles [9]. There is a lack of studies on trends in the prevalence of chronic diseases that allow differentiating between effects due to aging of the population and these other factors. Since most projections of the future number of people with one or more chronic diseases consider primarily the aging population, this may underestimate the real increase in chronic diseases and hence of the appropriate health services needed. More insight into the impact of the aging population and other factors would increase the precision of such projections in the future.

This study describes the trends in the prevalence of chronic diseases and multimorbidity in the Netherlands from 2001 till 2011, and the extent to which they can be ascribed to aging of the population. Estimates of these trends were derived from two types of population-based data: medical records from general practices and self-reported health status from health interview surveys. These sources offered a consistent window on trends in the general population by a continuous data collection and processing. Using both registration and self-reported data provides a more complete picture of trends in chronic diseases.

## Methods

### General practice registration

We used a longitudinal dataset based on electronic medical records of over 350,000 patients listed with one of the general practices that participate in the NIVEL Primary Care Database (formerly known as the Netherlands Information Network of General Practice (LINH)). This database comprises a representative sample of general practices distributed across the Netherlands [10]. All inhabitants are obligatory listed with a general practice, except for people living in nursing homes. Participating general practitioners routinely record data on consultations, including diagnoses, medication prescriptions and referrals to specialized care. Diagnoses are coded according to the International Classification of Primary Care, version 1 (ICPC-1). For this study only data from practices were used that met the criterion for completeness of registration: a percentage of valid ICPC coded diagnoses made during consultations of 60% or more.

The NIVEL Primary Care Database is an open network that allows practices and patients to join or stop at any time. Data from practices were included if data were available for at least two consecutive years. Therefore, the number of practices varied between 29 in 2005 and 105 in 2011. We included persons who were registered with the network at the beginning of any of the years 2004–2011 and at least one year prior to that year. The number of persons listed in the participating practices varied between 112,453 in 2006 and 359,682 in 2010 (mean = 229,723). The estimated disease prevalence was based on selected chronic conditions (see below) being registered as a diagnosis over a period of at least two years (the year of interest, the previous year, and if available, the year before that). A time window of two/three years was chosen in order to also capture those chronic conditions that do not require regularly visits to the general practitioner (GP) (for example, osteoarthritis or asthma). With this time window, general practice data from 2002 to 2011 are used to study trends between 2004 and 2011.

Twenty-eight of the most prevalent chronic diseases were selected in order to define the prevalence of chronic disease and multimorbidity (see S1 Table for the list of diseases and corresponding ICPC codes) [11, 12]. Multimorbidity was defined as the co-occurrence of two or more of the selected 28 diseases. From 2002 till 2009, these episodes were constructed by EPICON, an algorithm to group ICPC-coded contact records from electronic medical records in general practice into episodes of care [13]. Episodes of care include all patients contacts and all drug prescriptions pertaining to a specific health problem [14]. Thus two consultations for the same health problem are grouped into one episode of care. Consider, for instance, a patient who visits the general practitioner with a chronic cough, and a few months later the same patient is diagnosed with COPD. Most likely, both diagnoses refer to the same health problem and to avoid double counting the two diagnoses were grouped into one episode of care named COPD. The algorithm is designed to recognise different contacts and cluster them in one episode of care. The prevalence of a disease is therefore depending on the specifics in the algorithm. Since 2010, most GPs allocate patient contacts into episodes of care by themselves in the electronic medical records.

NIVEL Primary Care Database is registered with the Dutch Data Protection Authority; data are handled according to the data protection guidelines of the authority. According to Dutch legislation, studies using this kind of observational data do not require medical ethical approval. Data were provided in a way that no individual person can be identified.

### Health survey

Statistics Netherlands conducts continuous health surveys in which randomly chosen inhabitants are invited to answer questions regarding their health. Each year approximately 15,000 Dutch inhabitants are invited (before 2010 the sample was about 10,000 inhabitants). Until 2010, all respondents were interviewed at home and respondents of 12 years and older were additionally asked to complete a written questionnaire including questions on 16 health problems and chronic diseases. From 2010 onwards a mixed-mode design was applied: a web-based questionnaire for all ages with questions on general health and in case of non-response an interview by telephone or at home. A second web-based or written questionnaire was sent to respondents (of the first questionnaire) of 12 years and older including questions on health problems and chronic diseases. Circa 60–65% of the invited people responded to the first questionnaire and circa 55% of those responded to the second questionnaire. Statistics Netherlands adjusted the yearly samples for differences in composition with the Dutch population by applying a weighting factor based on sex, age, ethnicity, marital status, geographic characteristics, and survey season [15, 16]. Health Survey data from 2001 to 2011 were used because the questions on chronic diseases did not change during this period [17, 18]. We selected eleven prevalent chronic diseases (see S2 Table for the list of diseases) and these were considered if present during the last 12 months. We selected all respondents of 25 years and older, on average 5,400 persons each year in the period 2001–2011.

Ethics approval and informed consent was not required for the health survey data since it concerned only self-reported data. Data were provided in a way that no individual person can be identified. Details on data protection issues are described in the Quality Declaration of Statistics Netherlands, available at: http://www.cbs.nl/NR/rdonlyres/15241495-A800-460E-A740-FDC9B815D0A6/0/201401qualitydeclarationofstatisticsnetherlands.pdf

### Statistical analyses

Logistic regression analyses were applied to estimate the change in prevalence rates of having at least one chronic disease or multimorbidity over time and to test whether any time trend differed from zero. Stratified analyses were performed according to sex and age groups (25–54, 55–64, 65–74, ≥75 years, data on the age group 0–24 years was available only for the general practice data). To ensure similar analyses of time trends in both data sources, trends in the total population were limited to the population of 25 years and older. In order to distinguish the proportion of the trend that can be ascribed to aging of the population and proportion of the trend that can be ascribed to other factors, we performed trend analyses with and without standardisation of the prevalence estimates for the total population with the year 2011 as reference. Standardisation was performed based on the population size and distribution over 5-year age groups and sex in 2011 [19]. The difference between the non-standardised trend and the standardised trend is an indication of the effect of aging of the population on the rise in chronic diseases and multimorbidity. Therefore, proportions of the trend attributed to ageing of the population were determined by dividing the difference between the non-standardised trend and the standardised trend by the non-standardised trend.

Prevalence estimates in the general practice data were determined using multilevel analyses, to control for possible interpractice variation, clustering of observations within general practices, and the dependence between yearly measurements within patients. Analyses were further adjusted for length of registration of patients in the specific practice. General practice data were analyzed using MLwin V.2.02 software and health survey data were analyzed using R software. Statistical significance was defined as p<0.05.

A change in general practice recording from 2010 onwards (construction of episodes of care by an algorithm vs. by GP) could have improved registration quality. In addition, a disease management program based on bundled payments for diabetes mellitus was implemented during the study period and this reform may have contributed to better recording of the diagnosis of diabetes mellitus or enhanced case-finding. Therefore, we conducted two sensitivity analyses: 1) including a sample of general practices with the highest quality registration (75^th^ percentile of practices with the lowest number of missing ICPC-codes) 2) excluding diabetes mellitus from the list of chronic diseases (including 27 chronic diseases).

## Results

### Trends in chronic disease prevalence

**Fig 1:**
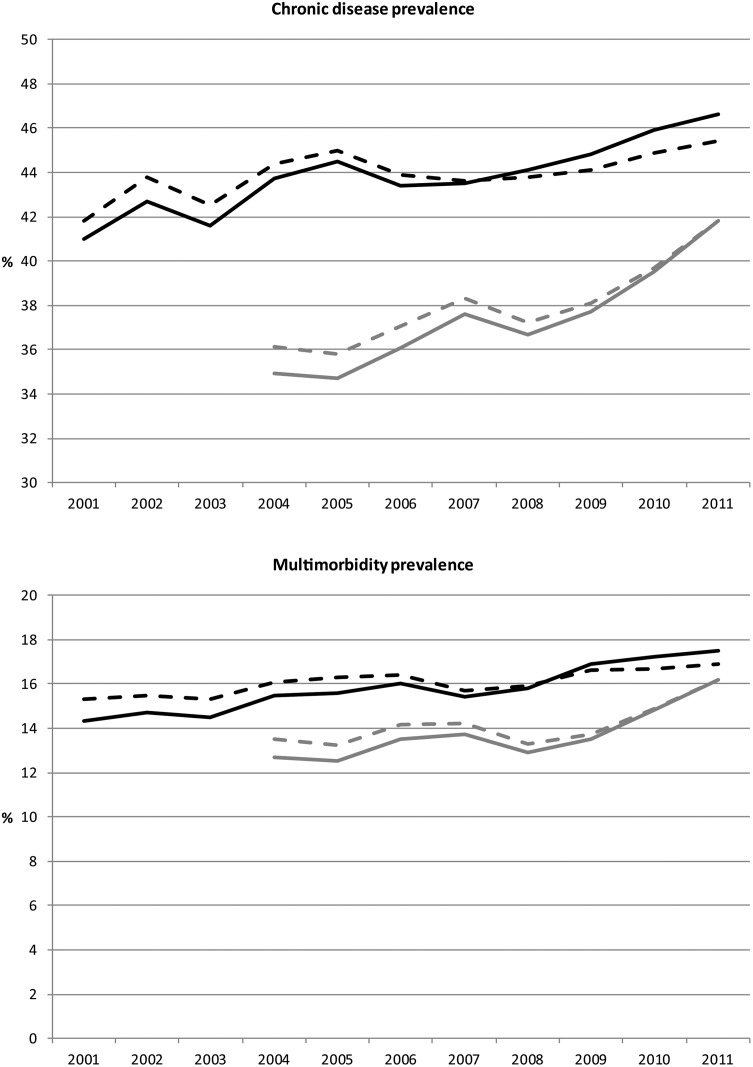
The prevalence of at least one chronic disease (upper Fig) and the prevalence of multimorbidity (lower Fig) in the general population of 25 years and older, over the period 2001–2011. Legend: grey solid line = GP registered diagnoses; grey dotted line = GP registered diagnoses, prevalence standardized for the Dutch population in 2011; black solid line = self-reported chronic diseases; black dotted line = self-reported chronic diseases, prevalence standardized for the Dutch population in 2011.

The prevalence of having at least one chronic disease based on a selection of 28-recorded diagnoses in general practice was 34.9% in 2004 and 41.8% in 2011, a statistically significant increase of 6.9 percentage points during the period 2004–2011 for the population of 25 years and older (Fig 1, Table 1). Standardisation to the population in 2011 reduced the increase to 5.7 percentage points, which was still a significant rise. The increase in prevalence was significant for men and women and for all age groups except for those aged 0–24 years. For all age groups over 25 years the increase was significantly larger in men than in women (p<0.01).

**Table 1:** Trends in the prevalence of diagnosed chronic diseases and multimorbidity in general practices over the period 2004–2011.

	**Modelled percentage persons with any chronic disease**								**Trends 2004–2011**		
	2004	2005	2006	2007	2008	2009	2010	2011	Crude change (perc. points ^2^)	Modelled change (% from 2004)	P ^3^
**Men and women**											
0–24 yrs	11.2	10.9	11.1	11.8	10.8	10.6	10.5	11.2	0.0	-3.6	0.29
25–54 yrs	24.3	23.8	24.5	26.4	25.2	26.1	26.7	28.4	4.1	16.7	**<0.01**
55–64 yrs	41.3	41.6	42.7	43.5	43.1	43.6	46.5	49.1	7.8	16.8	**<0.01**
65–74 yrs	56.3	56.4	58.5	60.0	58.3	58.9	61.7	65.0	8.7	13.0	**<0.01**
≥75 yrs	71.4	71.0	74.1	73.2	71.6	73.3	77.3	79.6	8.2	10.1	**<0.01**
Total ≥25 yrs	34.9	34.7	36.1	37.6	36.7	37.7	39.5	41.8	6.9	18.6	**<0.01**
Total std ≥25 yrs^1^	36.1	35.8	37.0	38.3	37.2	38.1	39.7	41.8	5.7	14.5	**<0.01**
**Men**											
0–24 yrs	10.8	10.6	10.7	11.3	10.3	10.0	9.9	10.6	-0.2	-6.2	0.07
25–54 yrs	20.5	19.8	20.5	22.2	21.5	22.2	22.7	24.3	3.8	19.2	**<0.01**
55–64 yrs	38.7	39.6	40.4	41.5	41.3	42.0	45.1	47.6	8.9	20.8	**<0.01**
65–74 yrs	54.0	54.2	56.9	58.8	57.4	58.5	61.0	64.7	10.7	17.6	**<0.01**
≥75 yrs	69.2	69.4	73.2	72.6	71.1	73.6	77.3	79.8	10.6	13.8	**<0.01**
Total ≥25 yrs	30.3	30.3	31.7	33.4	32.9	34.0	35.7	38.0	7.5	24.5	**<0.01**
Total std ≥25 yrs^1^	31.7	31.6	32.8	34.1	33.4	34.3	36.0	38.1	6.4	18.3	**<0.01**
**Women**											
0–24 yrs	11.7	11.2	11.5	12.3	11.2	11.1	11.1	11.9	0.2	-0.9	0.79
25–54 yrs	28.4	28.0	28.8	30.6	29.0	30.0	30.7	32.4	4.0	12.9	**<0.01**
55–64 yrs	44.0	43.8	45.0	45.4	44.9	45.3	47.8	50.6	6.6	12.8	**<0.01**
65–74 yrs	58.4	58.4	59.9	61.1	59.1	59.3	62.3	65.3	6.9	9.2	**<0.01**
≥75 yrs	72.8	72.1	74.7	73.6	72.0	73.2	77.3	79.5	6.7	7.9	**<0.01**
Total ≥25 yrs	39.3	39.1	40.4	41.7	40.4	41.3	43.2	45.4	6.1	13.9	**<0.01**
Total std ≥25 yrs^1^	40.3	39.9	41.1	42.2	40.8	41.5	43.3	45.4	5.1	11.2	**<0.01**
	**Modelled percentage persons with 2 or more chronic diseases**								**Trends 2004–2011**		
	2004	2005	2006	2007	2008	2009	2010	2011	Crude change (perc. points ^2^)	Modelled change (% from 2004)	P ^3^
**Men and women**											
0–24 yrs	0.6	0.6	0.6	0.7	0.6	0.7	0.7	0.8	0.2	35.1	**0.03**
25–54 yrs	5.7	5.5	5.9	6.2	5.5	5.7	6.0	6.6	0.9	11.3	**0.02**
55–64 yrs	14.2	14.3	15.0	15.3	14.5	15.0	16.4	18.3	4.1	23.6	**<0.01**
65–74 yrs	26.2	25.5	27.2	27.3	25.8	26.7	29.2	31.6	5.4	17.5	**<0.01**
≥75 yrs	42.8	41.8	45.1	43.6	41.6	43.2	47.6	50.4	7.8	15.0	**<0.01**
Total ≥25 yrs	12.7	12.5	13.5	13.7	12.9	13.5	14.8	16.2	3.5	23.9	**<0.01**
Total std ≥25 yrs^1^	13.5	13.2	14.1	14.2	13.3	13.7	14.9	16.2	2.7	16.4	**<0.01**
**Men**											
0–24 yrs	0.5	0.4	0.4	0.5	0.5	0.5	0.5	0.5	0.1	23.6	0.18
25–54 yrs	4.2	4.0	4.3	4.7	4.2	4.4	4.7	5.0	0.8	18.8	**<0.01**
55–64 yrs	12.6	12.5	12.9	13.4	13.2	13.9	15.3	17.3	4.7	35.2	**<0.01**
65–74 yrs	24.4	23.7	25.2	26.0	24.7	25.7	28.3	30.5	6.1	23.6	**<0.01**
≥75 yrs	39.5	39.7	43.0	41.9	40.4	43.1	47.2	50.2	10.7	23.9	**<0.01**
Total ≥25 yrs	10.1	10.0	10.8	11.3	10.8	11.5	12.8	14.0	3.9	37.1	**<0.01**
Total std ≥25 yrs^1^	11.0	10.8	11.5	11.9	11.2	11.8	12.9	14.0	3.0	25.1	**<0.01**
**Women**											
0–24 yrs	0.7	0.7	0.8	0.9	0.8	0.9	0.9	1.1	0.4	41.8	**<0.01**
25–54 yrs	7.4	7.2	7.5	7.7	6.8	7.1	7.4	8.1	0.8	5.4	0.21
55–64 yrs	15.9	16.2	17.3	17.2	15.8	16.1	17.5	19.4	3.5	13.9	**<0.01**
65–74 yrs	27.9	27.2	29.1	28.4	26.9	27.7	30.0	32.7	4.8	12.9	**<0.01**
≥75 yrs	44.9	43.1	46.4	44.7	42.4	43.2	47.9	50.6	5.7	10.2	**<0.01**
Total ≥25 yrs	15.3	15.1	16.1	16.0	14.9	15.4	16.8	18.3	3.0	14.7	**<0.01**
Total std ≥25 yrs^1^	15.9	15.6	16.6	16.4	15.2	15.6	16.9	18.3	2.4	10.2	**<0.01**

^1^ Total prevalence for men and women of 25 yrs and older, were standardised according to the age distribution of the population in 2011 (Statistics Netherlands).

^2^ Percentage points.

^3^ The P value was based on the modelled difference 2004 and 2011, adjusted for sex and age and clustering in GPs.

The prevalence based on self-reported chronic diseases was 41.0% in 2001 and 46.6% in 2011, a significant increase of 5.6 percentage points during the period 2001–2011 (Fig 1, Table 2). Standardisation to the population in 2011 showed a significant increase of 3.6 percentage points. The increase in prevalence was statistically significant for women, for men only the non-standardised (for age) increase was significant.

**Table 2:** Trends in the prevalence of self-reported chronic diseases and multimorbidity in health surveys over the period 2001–2011.

	**Crude percentage persons with any chronic disease**											**Trends 2001–2011**		
	2001	2002	2003	2004	2005	2006	2007	2008	2009	2010	2011	Crude change (perc. points ^2^)	Modelled change (% from 2001)	P ^3^
**Men and women**														
25–54 yrs	35.9	37.8	35.8	36.5	37.0	35.0	35.5	35.7	35.0	37.5	37.6	1.9	0.7	0.76
55–64 yrs	42.1	44.0	45.6	48.1	51.4	49.5	47.1	48.0	50.7	51.1	51.2	9.1	16.2	**<0.01**
65–74 yrs	54.0	53.3	51.5	58.7	58.3	57.9	56.8	56.6	57.0	56.6	57.5	3.5	6.2	0.05
≥75 yrs	64.0	64.7	63.9	67.6	64.4	67.6	70.1	68.3	70.6	65.6	68.8	4.8	7.3	**0.03**
Total	41.0	42.7	41.6	43.7	44.5	43.4	43.5	44.1	44.8	45.9	46.6	5.6	11.1	**<0.01**
Total std^1^	41.8	43.8	42.5	44.4	45.0	43.9	43.6	43.8	44.1	44.9	45.4	3.6	5.2	**0.02**
**Men**														
25–54 yrs	27.4	30.0	27.2	30.8	32.2	28.3	28.3	28.6	29.1	30.7	30.2	2.8	4.6	0.25
55–64 yrs	34.6	42.4	41.6	43.9	46.2	46.4	45.4	44.2	45.1	47.1	47.9	13.3	20.4	**<0.01**
65–74 yrs	50.8	45.2	41.9	56.1	51.3	52.0	49.7	49.7	50.2	49.6	55.8	5.0	10.3	**0.04**
≥75 yrs	59.6	60.1	50.6	59.7	56.9	63.2	59.0	60.9	61.5	54.7	59.7	0.1	1.9	0.75
Total	33.7	36.1	33.5	38.5	39.2	37.6	37.0	37.5	38.4	40.1	41.7	8.0	17.5	**<0.01**
Total std^1^	34.0	36.5	33.7	38.5	38.9	37.4	36.7	36.4	37.1	37.5	38.7	4.7	8.1	0.08
**Women**														
25–54 yrs	43.1	44.6	43.6	41.6	41.3	41.1	41.7	42.0	40.1	43.0	43.7	0.6	-2.4	0.38
55–64 yrs	50.0	45.6	49.7	52.7	56.7	52.9	48.8	51.7	56.2	55.5	54.5	4.5	12.7	**<0.01**
65–74 yrs	57.5	61.6	60.8	61.0	65.2	63.0	63.2	63.2	63.8	64.6	59.6	2.1	4.4	0.28
≥75 yrs	67.2	68.1	74.9	73.9	70.2	70.7	77.6	73.1	76.6	76.3	77.2	10.0	12.1	**<0.01**
Total	47.5	48.6	49.1	48.4	49.5	48.7	49.2	49.9	50.4	51.4	51.2	3.7	6.9	**<0.01**
Total std^1^	48.6	50.5	50.7	49.9	50.7	49.8	50.7	50.2	50.1	51.7	51.3	2.7	3.1	**<0.05**
	**Crude percentage persons with 2 or more chronic diseases**											**Trends 2001–2011**		
	2001	2002	2003	2004	2005	2006	2007	2008	2009	2010	2011	Crude change (perc. points ^2^)	Modelled change (% from 2001)	P ^3^
**Men and women**														
25–54 yrs	10.3	10.9	9.5	10.5	10.0	10.6	9.1	9.9	10.6	11.5	11.0	0.7	7.6	0.15
55–64 yrs	16.0	18.3	19.0	19.5	20.2	19.2	19.4	18.0	19.9	21.5	20.1	4.1	15.6	**0.02**
65–74 yrs	24.0	22.1	23.0	26.5	26.0	26.6	25.2	25.3	24.6	23.7	26.1	2.1	5.9	0.35
≥75 yrs	32.4	28.5	31.7	30.8	33.4	32.2	36.3	34.8	37.0	31.2	35.3	2.9	14.7	**0.05**
Total	14.3	14.7	14.5	15.5	15.6	16.0	15.4	15.8	16.9	17.2	17.5	3.2	21.6	**<0.01**
Total std^1^	15.3	15.5	15.3	16.1	16.3	16.4	15.7	15.9	16.6	16.7	16.9	1.6	9.3	**<0.01**
**Men**														
25–54 yrs	7.7	7.5	6.7	7.1	7.3	8.0	6.9	7.1	7.3	8.8	7.0	-0.7	4.8	0.60
55–64 yrs	11.1	16.9	17.0	14.5	16.3	16.5	18.2	15.5	15.6	18.5	17.7	6.6	24.6	**0.02**
65–74 yrs	19.2	13.8	16.2	21.1	22.0	19.7	21.2	19.8	20.0	18.7	22.5	2.3	20.6	0.06
≥75 yrs	24.8	23.2	18.6	23.6	28.6	28.5	28.3	25.0	24.4	23.6	24.1	-0.7	5.1	0.69
Total	10.8	11.0	10.7	11.3	12.5	12.6	12.7	12.0	12.4	14.2	13.8	3.0	30.2	**<0.01**
Total std^1^	11.0	11.4	10.9	11.4	12.5	12.6	12.5	11.7	11.8	12.7	12.1	1.1	11.2	**0.03**
**Women**														
25–54 yrs	12.5	13.8	12.0	13.5	12.4	13.0	11.0	12.4	13.4	13.7	14.4	1.9	7.5	0.23
55–64 yrs	21.2	19.7	21.1	25.0	24.3	22.3	20.6	20.4	24.3	24.8	22.5	1.3	9.2	0.26
65–74 yrs	29.1	30.4	29.7	31.4	30.0	32.7	28.8	30.7	29.3	29.7	30.6	1.5	-0.1	0.99
≥75 yrs	38.0	32.4	42.4	36.6	37.3	34.8	41.8	41.2	45.3	39.7	45.7	7.7	23.5	**<0.01**
Total	17.4	18.1	18.0	19.3	18.6	19.0	17.8	19.1	20.9	20.1	21.0	3.6	17.2	**<0.01**
Total std^1^	19.3	19.3	19.5	20.6	20.0	19.9	18.8	19.8	21.0	20.7	21.5	2.2	8.5	**0.03**

^1^ Total prevalences for men and women, men, and women, standardised according to the age distribution in 2011 and retrieved from the model.

^2^ Percentage points.

^3^ The P value was based on the modelled difference between 2001 and 2011.

### Trends in multimorbidity prevalence

The prevalence of multimorbidity based on general practice recorded diagnoses was 12.7% in 2004 and 16.2% in 2011, a significant increase of 3.5 percentage points (Fig 1, Table 1). Standardisation to the population in 2011 showed a significant increase of 2.7 percentage points. The increase in prevalence was significant for men and women and for all age groups except for men aged 0–24 years and women aged 25–54 years. For all age groups over 25 years the increase was significantly larger in men compared to women (p<0.01).

Multimorbidity prevalence based on self-reported chronic diseases was 14.3% in 2001 and 17.5% in 2011, a significant increase of 3.2 percentage points (Fig 1, Table 2). Standardisation to the population in 2011 reduced the increase to 1.6 percentage points. The increase in prevalence was significant for men, women, and the age groups of 55–64 and over 75 years.

### Proportion of trends attributed to aging of the population

About one fifth of the trend in diagnosed chronic diseases and the trend in multimorbidity recorded in general practice was attributed to aging of the population (Table 3). The health survey data showed a higher proportion of the trend attributed to aging: about one third for the trend in at least one chronic disease, and half of the trend for multimorbidity.

**Table 3:** Proportion of the trend in the prevalence of chronic diseases and multimorbidity attributed to ageing of the population, retrieved from general practices over the period 2004–2011 and health surveys over the period 2001–2011.

	Proportion of trend attributed to ageing of the population^1^	
	General practice	Health surveys
**Any chronic disease**		
Men and women	0.17	0.36
Men	0.15	0.41
Women	0.16	0.27
**Multimorbidity**		
Men and women	0.23	0.50
Men	0.23	0.63
Women	0.20	0.39

^1^ Proportions represent an indication of the effect of aging of the population on the rise in chronic diseases and multimorbidity. Proportions were derived at by dividing the total crude change—total crude change standardized / total crude change (based on the data in Tables 1 and 2).

Applying the two sensitivity analyses did not alter the findings for trends based on the general practice registered diagnoses.

## Discussion

Our findings show a rise in the prevalence of chronic diseases and multimorbidity since the beginning of this century. This trend was only partially explained by aging of the population, implying that other epidemiological, medical and societal developments or circumstances explain a substantial part of this rising trend. The increase in prevalence was observed on the basis of recorded diagnoses of chronic diseases in general practice and on the basis of self-reported chronic diseases in a national health survey. Age groups over 55 years of age showed the largest increase. In most age-sex groups, trends as observed in data from the health survey were less marked than in the general practice data.

The (main) strength of our study is that trend analyses were conducted in two different types of datasets over nearly the same period. Everyone in the Netherlands is registered within a general practice, and the general practice population and general practices are representative for the Dutch population and the Dutch general practitioners, respectively [20]. Moreover, standardised recording procedures were used. General practices not fulfilling the criteria for completeness of the registration were excluded from the analyses since incomplete data on ICPC codes lead to underestimation of chronic disease prevalence. Though exclusion of these practices may have caused minimal selection bias, a general practice database is still the most representative source for prevalence of chronic disease in the Netherlands because all Dutch inhabitants are obligatory listed in a general practice. Response in health surveys is always selective but an advantage of this dataset is that it also includes diseases that do not require regular general practitioner visits, such as asthma. The different nature of both datasets is illustrated by a 5% higher chronic disease prevalence based on self-reported chronic diseases compared to GP diagnosed chronic diseases, despite the much smaller selection of diseases in the self-reported dataset. A limitation of both data sources is that a small group of elderly in nursing homes is not included (<1% of the Dutch population)[21]. The effect is that the prevalence of chronic diseases in the general population is slightly underestimated. However, time trends are minimally affected since the number of elderly in nursing homes is very small.

The method of direct standardisation does not take into account age differences within 5-year age categories. As additional sensitivity analysis we performed logistic regression with age adjustment. Overall, the results confirmed the rise in the prevalence of chronic diseases and multimorbidity since the beginning of this century, especially in the higher age groups. One difference is that increasing trends in the prevalence of multimorbidity for women were not all confirmed in both datasets, but for the total group of women (based on data from general practices) a significant trend in the prevalence of multimorbidity was still observed. Although the reported rise in prevalence was already smaller in women than in men, it may also suggest that factors contributing to the rise in prevalence of multimorbidity may differently affect men and women.

Some changes in registration procedures could have affected the results of this study. Recording procedures regarding episodes of care in general practices improve continuously. We conducted a sensitivity analysis including practices in the highest quartile of recording quality for each year, which showed similar trends in chronic diseases and multimorbidity. This may imply that the increase in prevalence rates is not a registration artefact; however, it may also imply that even in the best-recording practices the quality of the recording has been improving. It should be further noted that the increase in prevalence in the general practice based data was most substantial in 2010 and 2011. During this period a disease management program for diabetes was introduced in Dutch general practices with corresponding changes in the payment system. Our concern was that this reform would lead to better recording of the diagnosis of diabetes or enhanced case-finding, but sensitivity analyses including all chronic diseases except diabetes showed similar trends. How the prevalence of other chronic diseases is affected by the introduction of the disease management program for diabetes is unknown: patients visit their care providers more often which may lead to earlier diagnosis of other chronic diseases, but prevalence may also decrease because interventions take into account comorbidities that share common risk factors.

Also with regard to the health survey there were changes in procedures. Data collection changed in 2010 from face-to-face interviews to a two-stage approach, starting with internet-based questionnaires. According to a report of Statistics Netherlands this did not affect response rates or results [22]. The lines representing the prevalence of one or more chronic diseases from 2001 to 2011 (Fig 1) do not demonstrate a discontinuity at the year 2010. A final point is that the number of people of 80 years and older is increasing in populations. The age distributions within the age category of 75 years and older may be heterogeneous. Therefore, future studies may divide the age category of 75 years and older in smaller age categories to take this into account.

Only few studies on general trends (not disease-specific) in the prevalence of chronic diseases and multimorbidity are available and most of them considered a total trend not distinguishing effects attributed to aging of the population and other developments [4, 5]. Comparison of such trend data is hampered due to large differences in the diseases included, in type and characteristics of the datasets, in standardisation for sex and age, and in the country or period of study [23, 24]. Nevertheless, two studies retrieved show similar results to our study, a clear increase in the prevalence of chronic diseases and multimorbidity in the past 25 years, that cannot be explained by the aging of the population [25, 26]. A study in one Dutch region using general practice based data (different from those presented here), showed a rise in the prevalence of patients registered with one or more chronic diseases in the period 1985–2005 [26]. A Swedish study showed a rise in the prevalence of conditions from 25% in 1992 to 31% in 2002, based on 14 conditions among persons of 77 years and older [25]. This Swedish study also showed a rise in multiple conditions from 10% in 1992 to 15% in 2002. Trends are larger in men compared to women, which was also shown by Freid et al. [4]. A larger increase in prevalence for men may be explained by sex differences in trends in individual chronic diseases and by the relation between the diagnosis of chronic diseases and health care utilisation: the more contact with health care providers the higher the chance that chronic diseases are diagnosed in an early stage. Traditionally, women have a higher health care utilisation [27]. So, the larger increase in the prevalence of chronic diseases and multimorbidity for men may reflect a catch-up in health care utilisation of men compared to women.

This study shows an increasing trend in the prevalence of chronic diseases and multimorbidity differentiating between a part that is due to the aging of the population and a part that is due to other developments or circumstances. Our figures show that aging of the population only explained a part of the prevalence increase between 2001–2011. Thus, other factors contribute to the rise in prevalence. First, improved case finding through better detection might result in an increase in the proportion of diagnosed cases. This, of course, does not affect the ‘real’ prevalence. Improved treatment of diseases and their risk factors might result in an increased probability of survival for those with chronic diseases. Both factors lead to an increase in the duration of the period persons live with chronic diseases. Second, changes in several lifestyle and environmental risk factors for chronic diseases contribute to increased occurrence (incidence) of chronic diseases [9]. Both the prevalence and the lifetime exposure to overweight and obesity have risen [28] leading to increased incidence of diabetes, cancer, and cardiovascular disease. Third, several factors related to general circumstances in society may affect chronic disease prevalence. The 24/7 economy and the global financial crisis have led to high demands that may affect health and health care utilisation [29, 30]. Over the past 30 years, the use of health care facilities in the Netherlands has obviously increased [31]. People visit medical professionals regularly and therefore more and earlier disease diagnosing is likely. Fourth, self-reporting chronic diseases may have increased in recent decades [32]. Most likely people are nowadays more aware of their chronic diseases, but this explanation regards only the observed trends in the health survey data. Fifth, as earlier mentioned, continuous improvement of recording procedures affect the estimated prevalence of chronic diseases in general practice based data. This factor may also explain the discrepancy between the proportions of the trend attributed to aging of the population from general practice data and health surveys. Recorded data on chronic diseases seem more sensitive to other developments than self-reported chronic diseases. The exact role and contribution of these different explanations for the rise in chronic diseases and multimorbidity over the past decade is unknown. Aforementioned explanations are hypothetical and may be incomplete, and evidence for the association between these developments and circumstances and the increased trend in the prevalence of chronic diseases is lacking.

Both aging of the population and other developments or circumstances affect the prevalence of chronic diseases and multimorbidity. As projections of the future number of people with one or more chronic diseases consider primarily the aging population, the real increase in chronic diseases may be underestimated. Therefore, projections of the future number of people with one or more chronic diseases should consider other driving forces besides changing of the age structure of the population. With more people with chronic diseases and multimorbidity, a growing need for treatment, monitoring and management of diseases seems likely, which leads to a higher healthcare burden [33–35]. Considering this, the rise in prevalence has enormous consequences for the organisation and capacity of health services.

## Supporting Information

S1 Table Selection of chronic diseases in the general practice registration (NIVEL Primary Care Database) (including ICPC codes). (DOCX)

S2 Table Selection of chronic diseases in the health surveys of Statistics Netherlands. (DOCX)
