# Supplementary material for: Time Trends in Prevalence of Chronic Diseases and Multimorbidity Not Only due to Aging: Data from General Practices and Health Surveys
Source: PLoS One. 2016 Aug 2;11(8):e0160264. doi: 10.1371/journal.pone.0160264 (PMC4970764; doi:10.1371/journal.pone.0160264)
Supplement: S1 Table — (DOCX) [file pone.0160264.s001.docx]

| **Selection of chronic diseases in general practice registration** | **ICPC codes** |
| --- | --- |
| HIV/AIDS | B90 |
| Cancer | A79, B72, B73, B74, D74, D75, D76, D77, L71, N74, R84, R85, S77, T71, U75, U76, U77, W72, X75, X76, X77, Y77, Y78 |
| Visual disorder | F84, F92-F93, R83 |
| Hearing disorder | H84-H86 |
| Congenital cardiovascular anomaly | K73 |
| Heart valve disease | K83 |
| Heart failure | K77 |
| Coronary heart disease | K74-K76 |
| Cardiac arrhythmia | K78-K80 |
| Stroke | K89-K90 |
| Reumatoid arthritis | L88 |
| Peripheral osteoarthrosis | L89-L91 |
| Chronic back or neck disorder | L83-L84, L86 |
| Osteoporosis | L95 |
| Parkinson’s disease | N87 |
| Epilepsy | N88 |
| Migraine | N89 |
| Chronic alcohol abuse | P15 |
| Dementia | P70 |
| Schizophrenia | P72 |
| Mood disorders | P73, P76 |
| Anxiety disorder | P74 |
| Burn-out (surmenage) | P78 |
| Personality disorder | P80 |
| Mental retardation | P85 |
| COPD | R91, R95 |
| Asthma | R96 |
| Diabetes Mellitus | T90 |
