# Supplementary material for: Time Trends in Prevalence of Chronic Diseases and Multimorbidity Not Only due to Aging: Data from General Practices and Health Surveys
Source: PLoS One. 2016 Aug 2;11(8):e0160264. doi: 10.1371/journal.pone.0160264 (PMC4970764; doi:10.1371/journal.pone.0160264)
Supplement: S2 Table — (DOCX) [file pone.0160264.s002.docx]

| **Health survey selection of chronic diseases** |
| --- |
| Cancer |
| Heart failure |
| Coronary heart disease |
| Migraine |
| Stroke |
| Reumatoid arthritis |
| Peripheral osteoarthrosis |
| Chronic back or neck disorder |
| Chronic intestinal disease |
| Asthma or COPD |
| Diabetes mellitus |
